# From Starvation to Neurological Crisis: A Case of Wernicke’s Encephalopathy Triggered by Laxative Abuse

**DOI:** 10.7759/cureus.78382

**Published:** 2025-02-02

**Authors:** Maryana Stryelkina, Britney Constans, Josh Alger, Kendahl Lyle, Aneesh George

**Affiliations:** 1 Internal Medicine, Baylor Scott & White Medical Center - Temple, Temple, USA; 2 Medical School, Texas A&M University College of Medicine, Temple, USA; 3 Internal Medicine, Central Texas Veterans Health Care System, Temple, USA

**Keywords:** anorexia nervosa (an), laxative abuse, non-alcoholic wernicke encephalopathy, thiamine, wernicke encephalopathy

## Abstract

Wernicke's encephalopathy (WE) is an acute neuropsychiatric disorder resulting from thiamine deficiency, most commonly associated with chronic alcohol use, but it can also arise from rare nonalcoholic etiologies such as anorexia nervosa. This report describes a case of WE in a 56-year-old female patient with anorexia nervosa and chronic laxative misuse, presenting with altered mental status, disorientation, visual disturbances, and ataxia. Clinical evaluation revealed severe malnutrition (BMI 15.0 kg/m²) and multiple electrolyte abnormalities, while MRI findings demonstrated signal abnormalities in the bilateral medial thalamic and periaqueductal gray matter, consistent with WE. Chronic laxative abuse exacerbated thiamine depletion, leading to the development of WE. The patient was treated with high-dose intravenous thiamine and nutritional rehabilitation, resulting in complete recovery of neurological function within five days. This case emphasizes the importance of considering WE in malnourished patients presenting with encephalopathy, even in the absence of alcohol use, and highlights the critical role of early diagnosis, prompt thiamine replacement, and nutritional support in preventing irreversible neurological damage and ensuring recovery.

## Introduction

Wernicke’s encephalopathy (WE) is a potentially fatal neuropsychiatric syndrome caused by thiamine deficiency. While classically associated with chronic alcohol use disorder, nonalcoholic etiologies are increasingly recognized, particularly in conditions associated with malnutrition or impaired thiamine absorption [1-3]. Thiamine is a critical cofactor in enzymatic pathways involved in glucose metabolism and energy production [4]. Its deficiency can lead to cellular energy failure, oxidative stress, and subsequent neuronal injury, particularly in metabolically active brain regions such as the mammillary bodies, medial thalami, and periaqueductal gray matter [5,6].

The clinical presentation of WE is variable and often nonspecific. The classical triad of encephalopathy, oculomotor dysfunction, and ataxia is observed in less than 10% of cases, contributing to frequent delayed diagnosis [7]. In the absence of timely intervention, WE may progress to irreversible neurological deficits, coma, or death [8]. Early recognition and prompt treatment with thiamine are critical to prevent permanent sequelae [9].

Anorexia nervosa, a severe psychiatric disorder characterized by self-imposed caloric restriction, an intense fear of weight gain, and a distorted body image, is a significant risk factor for malnutrition and its complications [10]. Chronic laxative abuse, a common behavior in individuals with eating disorders, further exacerbates nutritional deficiencies by inducing fluid and electrolyte imbalances, impaired nutrient absorption, and depletion of essential vitamins, including thiamine [11]. Despite these known risks, the incidence of Wernicke’s encephalopathy in the context of anorexia nervosa and laxative abuse remains a rare but underreported phenomenon, with limited representation in the medical literature. A systematic review by Oudman et al. analyzed 586 cases of nonalcoholic Wernicke-Korsakoff syndrome and found that approximately 2% were associated with anorexia nervosa, highlighting the rarity of WE in this population [2].

This case report describes a 56-year-old female with a history of anorexia nervosa who developed WE due to acute-on-chronic laxative abuse and severe malnutrition. This case underscores the importance of considering WE in malnourished patients presenting with altered mental status, even in nonalcoholic settings. We aim to emphasize the need for heightened clinical awareness, early diagnosis, and prompt management of WE in atypical presentations to prevent significant morbidity and mortality. 

## Case presentation

A 56-year-old female presented to the emergency department with altered mental status, visual disturbances, and difficulty walking. Her family reported progressive confusion and unsteady gait over the preceding week. On initial evaluation, she was disoriented (alert but oriented to person only), with slowed speech and difficulty maintaining attention. Neurological examination revealed impaired smooth pursuit eye movements, horizontal nystagmus, and ataxia, including inability to perform finger-to-nose or heel-to-shin testing. Her gait was wide-based and unsteady, requiring assistance to stand. 

Physical examination revealed a cachectic, malnourished female with a weight of 40.9 kg and a body mass index (BMI) of 15.0 kg/m², consistent with severe protein-energy malnutrition. Vital signs revealed sinus tachycardia with a heart rate of 101 bpm and a blood pressure of 102/67 mmHg, within the low-normal range. Laboratory studies revealed hypokalemia (2.8 mmol/L), hypophosphatemia (1.8 mg/dL), and a reduced folate level (1.7 ng/mL). Vitamin B12 levels were within the normal range (595 pg/mL). A summary of the patient’s laboratory findings is presented in Table 1.

**Table 1:** Key laboratory results and reference ranges

Laboratory Parameters	Patient Values	Status	Reference Ranges	Units
Potassium (K⁺)	2.8	Low	3.5 - 5.0	mmol/L
Phosphate (PO₄³⁻)	1.8	Low	2.5 - 4.5	mg/dL
Folate	1.7	Low	>4.0	ng/mL
Vitamin B12	595.0	Normal	200 - 900	pg/mL
Calcium (Ca²⁺)	8.1	Low	8.5 - 10.2	mg/dL
Chloride (Cl⁻)	105.0	Normal	96 - 106	mmol/L
Sodium (Na⁺)	139.0	Normal	135 - 145	mmol/L
Bicarbonate (HCO₃⁻)	21.0	Low	22 - 28	mmol/L

**Figure 1:**
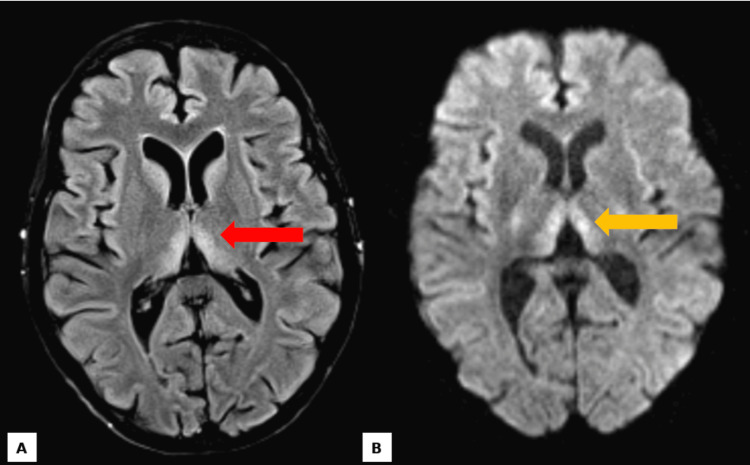
MRI Findings T2-weighted axial image (A, red arrow) demonstrating bilateral and symmetric hyperintense signal alterations localized to the medial portions of the thalami. Axial diffusion-weighted imaging (DWI) (B, yellow arrow) illustrating hyperintensities in the bilateral thalami.

Magnetic resonance imaging (MRI) of the brain demonstrated bilateral and symmetric hyperintensities in the medial thalami and periaqueductal gray matter on T2-weighted and fluid-attenuated inversion recovery (FLAIR) imaging, findings highly suggestive of Wernicke’s encephalopathy (Figure 1). The T2-weighted axial image (Figure 1A) highlights these signal alterations in the medial thalami, a region particularly vulnerable to thiamine deficiency-related damage. Additionally, axial diffusion-weighted imaging (DWI) (Figure 1B) reveals hyperintensities in the bilateral thalami, further reinforcing the diagnosis. Further history revealed a four-year history of anorexia nervosa, characterized by body image distortion, an extreme fear of weight gain, denial of hunger, and chronic laxative abuse. Two weeks prior to admission, she developed acute-on-chronic exacerbation due to excessive Lasix (furosemide) use (40 mg, unknown frequency), leading to a 5-pound weight loss, further exacerbating her nutritional deficiencies.

The patient was started on high-dose intravenous thiamine, leading to rapid and significant improvement. By the third day of hospitalization, her mental status improved markedly, with disorientation resolved and coordination partially restored. By the fifth day, she was ambulating with minimal assistance and had fully regained her baseline cognitive function.

## Discussion

WE is a well-documented neurologic emergency, yet it remains underdiagnosed, particularly in nonalcoholic settings. This case of WE in the context of anorexia nervosa with chronic laxative abuse highlights several important clinical considerations, including atypical etiologies, diagnostic challenges, and the importance of early intervention to prevent irreversible sequelae.

WE is a well-recognized neurologic emergency, yet it remains underdiagnosed, particularly in nonalcoholic contexts. Although chronic alcohol use disorder is the predominant cause, nonalcoholic etiologies, particularly those associated with malnutrition and impaired thiamine absorption, are increasingly identified [1-3]. This case represents a rare presentation, highlighting the need for increased clinical awareness in nontraditional contexts. The likely cause of thiamine deficiency in this patient was driven by inadequate dietary intake due to anorexia nervosa and the multifaceted effects of chronic laxative abuse. Laxative use accelerates gastrointestinal transit, potentially leading to impaired nutrient absorption in the small intestine, where thiamine is primarily absorbed [12-14]. Laxative-induced diarrhea further exacerbates deficiency through significant fluid and electrolyte losses, increasing metabolic stress and thiamine demand [15]. Additionally, chronic laxative use disrupts gut microbiota, reducing thiamine-producing bacteria and further depleting thiamine stores [16]. These effects, along with baseline malnutrition associated with anorexia nervosa, create a cycle of impaired absorption, increased demand, and worsening deficiency, increasing the risk of severe thiamine deficiency and development of WE.

WE frequently remains underdiagnosed due to its nonspecific clinical presentation. The classic triad of encephalopathy, oculomotor dysfunction, and ataxia are uncommon, often resulting in delayed recognition [7,17]. In this case, the patient exhibited two hallmark features: encephalopathy and ataxia-along with nystagmus, a nonspecific but commonly observed finding in WE. The overlap of these symptoms with other conditions, particularly in malnourished individuals with coexisting psychiatric disorders, underscores the necessity of maintaining a high index of clinical suspicion [18]. Neuroimaging is integral to diagnostic confirmation, as evidenced by this case, where MRI demonstrated symmetric hyperintensities in the medial thalami and periaqueductal gray matter, findings characteristic of WE [19]. These imaging findings highlight the critical role of advanced diagnostic modalities in ensuring timely diagnosis and intervention, particularly in atypical or nonalcoholic presentations. 

Early recognition and treatment with high-dose intravenous thiamine are essential to prevent irreversible neurological damage in WE. In this case, the patient’s rapid recovery following timely intervention highlights the condition’s reversibility with appropriate management. Intravenous thiamine, with its superior bioavailability, is the preferred treatment for severe deficiency, ensuring effective restoration of thiamine levels [20].

This case highlights the growing recognition of nonalcoholic WE, particularly in the setting of anorexia nervosa and acute-on-chronic laxative abuse, emphasizing the critical importance of early diagnosis and intervention to prevent disease progression and improve outcomes. Given the high risk of delayed diagnosis in such cases, clinicians should maintain a high index of suspicion when evaluating malnourished patients with altered mental status. Future studies should focus on developing standardized screening protocols for early detection in at-risk populations, ultimately enhancing timely recognition and treatment of WE.

## Conclusions

This case contributes to the growing body of literature emphasizing the diverse etiologies of WE and the critical need for increased clinical awareness in atypical presentations. Anorexia nervosa remains a significant but underrepresented risk factor for WE, particularly when compounded by behaviors such as chronic laxative abuse. Increased recognition of this association can facilitate earlier diagnosis and intervention, ultimately improving outcomes for this vulnerable population. 

## References

[1] Hutcheon DA (2015). Malnutrition-induced Wernicke's encephalopathy following a water-only fasting diet. Nutr Clin Pract.

[2] Oudman E, Wijnia JW, Oey MJ, van Dam M, Postma A (2021). Wernicke-Korsakoff syndrome despite no alcohol abuse: a summary of systematic reports. J Neurol Sci.

[3] McCormick LM, Buchanan JR, Onwuameze OE, Pierson RK, Paradiso S (2011). Beyond alcoholism: Wernicke-Korsakoff syndrome in patients with psychiatric disorders. Cogn Behav Neurol.

[4] Mrowicka M, Mrowicki J, Dragan G, Majsterek I (2023). The importance of thiamine (vitamin B1) in humans. Biosci Rep.

[5] Butterworth RF (2003). Thiamin deficiency and brain disorders. Nutr Res Rev.

[6] Desjardins P, Butterworth RF (2005). Role of mitochondrial dysfunction and oxidative stress in the pathogenesis of selective neuronal loss in Wernicke's encephalopathy. Mol Neurobiol.

[7] Sinha S, Kataria A, Kolla BP, Thusius N, Loukianova LL (2019). Wernicke encephalopathy-clinical pearls. Mayo Clin Proc.

[8] Sakurai K, Sasaki S, Hara M, Yamawaki T, Shibamoto Y (2009). Wernicke's encephalopathy with cortical abnormalities: clinicoradiological features: report of 3 new cases and review of the literature. Eur Neurol.

[9] Novo-Veleiro I, Mateos-Díaz AM, Rosón-Hernández B (2023). Treatment variability and its relationships to outcomes among patients with Wernicke's encephalopathy: a multicenter retrospective study. Drug Alcohol Depend.

[10] Attia E (2010). Anorexia nervosa: current status and future directions. Annu Rev Med.

[11] Roerig JL, Steffen KJ, Mitchell JE, Zunker C (2010). Laxative abuse: epidemiology, diagnosis and management. Drugs.

[12] Said HM (2011). Intestinal absorption of water-soluble vitamins in health and disease. Biochem J.

[13] Reidling JC, Lambrecht N, Kassir M, Said HM (2010). Impaired intestinal vitamin B1 (thiamin) uptake in thiamin transporter-2-deficient mice. Gastroenterology.

[14] Laforenza U, Patrini C, Alvisi C, Faelli A, Licandro A, Rindi G (1997). Thiamine uptake in human intestinal biopsy specimens, including observations from a patient with acute thiamine deficiency. Am J Clin Nutr.

[15] Marrs C, Lonsdale D (2021). Hiding in plain sight: modern thiamine deficiency. Cells.

[16] Igudesman D, Abbaspour A, Reed KK (2023). Laxative abuse is associated with a depleted gut microbial community structure among women and men with binge-eating disorder or bulimia nervosa: the binge eating genetics initiative. Psychosom Med.

[17] Kohnke S, Meek CL (2021). Don't seek, don't find: the diagnostic challenge of Wernicke's encephalopathy. Ann Clin Biochem.

[18] Eva L, Brehar FM, Florian IA (2023). Neuropsychiatric and neuropsychological aspects of alcohol-related cognitive disorders: an in-depth review of Wernicke's encephalopathy and Korsakoff's syndrome. J Clin Med.

[19] Manzo G, De Gennaro A, Cozzolino A, Serino A, Fenza G, Manto A (2014). MR imaging findings in alcoholic and nonalcoholic acute Wernicke's encephalopathy: a review. Biomed Res Int.

[20] Cantu-Weinstein A, Branning R, Alamir M, Weleff J, Do M, Nero N, Anand A (2024). Diagnosis and treatment of Wernicke's encephalopathy: a systematic literature review. Gen Hosp Psychiatry.

